# Early cortical specialization for face-to-face communication in human infants

**DOI:** 10.1098/rspb.2008.0986

**Published:** 2008-08-28

**Authors:** Tobias Grossmann, Mark H Johnson, Sarah Lloyd-Fox, Anna Blasi, Fani Deligianni, Clare Elwell, Gergely Csibra

**Affiliations:** 1 Centre for Brain and Cognitive Development, School of Psychology, Birkbeck, University of LondonMalet Street, London WC1E 7HX, UK; 2 Biomedical Optics Research Laboratory, Department of Medical Physics and Bioengineering, University College LondonMalet Place Engineering Building, Gower Street, London WC1E 6BT, UK

**Keywords:** face, eye gaze, infancy, communication, neuroimaging, social brain

## Abstract

This study examined the brain bases of early human social cognitive abilities. Specifically, we investigated whether cortical regions implicated in adults' perception of facial communication signals are functionally active in early human development. Four-month-old infants watched two kinds of dynamic scenarios in which a face either established mutual gaze or averted its gaze, both of which were followed by an eyebrow raise with accompanying smile. Haemodynamic responses were measured by near-infrared spectroscopy, permitting spatial localization of brain activation (experiment 1), and gamma-band oscillatory brain activity was analysed from electroencephalography to provide temporal information about the underlying cortical processes (experiment 2). The results revealed that perceiving facial communication signals activates areas in the infant temporal and prefrontal cortex that correspond to the brain regions implicated in these processes in adults. In addition, mutual gaze itself, and the eyebrow raise with accompanying smile in the context of mutual gaze, produce similar cortical activations. This pattern of results suggests an early specialization of the cortical network involved in the perception of facial communication cues, which is essential for infants' interactions with, and learning from, others.

## 1. Introduction

Humans are intensely social creatures (Herrmann *et al.* 2007). One major function of our brain is to enable us to recognize, manipulate and behave with respect to information about other humans. Much research on how the adult human brain processes the social world has shown that there is a network of specific brain areas, also called the social brain, preferentially involved during social perception and interaction (for reviews, see Adolphs 2003; Frith 2007). However, we have only just begun to understand when and how the capacities of the brain to read others' social behaviour emerge. It is thus pivotal to examine when in development cortical regions implicated in social processes in adults first become functionally active by studying the earliest stage of postnatal development, i.e. infancy.

The face as a prototypical social stimulus provides a wealth of relevant information. From birth, human infants preferentially orient towards face stimuli, especially when they include gaze-relevant contrast information (Johnson *et al.* 1991; Farroni *et al.* 2005). Among the social cues contained in the face, eye gaze plays a fundamental role in non-verbal social communication (Emery 2000). Compared with other primate species, human eyes are unique in their morphology because they have a widely exposed white sclera surrounding the darker iris, making it easy to discern the target of another person's attention during face-to-face interactions (Kobayashi & Kohshima 1997, 2001; Tomasello *et al.* 2006). It is therefore not surprising that the sensitivity to eyes and eye gaze is evident very early in ontogeny: newborns not only prefer to look at faces that have open eyes (Batki *et al.* 2000), but also exhibit a strong tendency to attend to faces that engage them in mutual gaze when compared with averted gaze (Farroni *et al.* 2002). Importantly, it has been argued that an early sensitivity to eye gaze serves as a major foundation for later development of social skills (Baron-Cohen 1995; Csibra & Gergely 2006). Indeed, an impairment of the sensitivity to eye gaze in general, and mutual gaze in particular, might be one of the early signs of atypical social development manifested in neurodevelopmental disorders such as autism (Phillips *et al.* 1992; Zwaigenbaum *et al.* 2005).

Face perception is mediated by a complex distributed neural system in humans. The functional organization of this system is characterized by a distinction between (i) the representation of invariant structural aspects of faces, which constitutes the basis of recognizing individuals, and (ii) the interpretation of dynamic changes of faces such as eye gaze and expression that are used in face-to-face communication with others (Haxby *et al.* 2000, 2002; Hoffman & Haxby 2000). The cortical brain regions most consistently activated during eye gaze processing in functional magnetic resonance imaging (fMRI) studies with adults are (i) the posterior superior temporal sulcus (STS), which is more generally implicated in the visual analysis of biological motion, facial expressions and human action (Allison *et al.* 2000), and (ii) the medial prefrontal cortex (MPFC), which is involved in various ‘mentalizing’ processes, especially those related to reading communicative intentions (Amodio & Frith 2006; Frith 2007; see Calder *et al.* (2007) for a discussion on which other brain structures might also be involved in eye gaze processing).

Mutual gaze (eye contact) serves as an important signal in face-to-face interactions that helps establish a communicative link between two people, and successful communication crucially depends on the ability to detect the intention to communicate (Frith 2007). Detecting mutual gaze (i.e. seeing a person shifting his gaze towards the viewer) evokes greater activity in the right posterior STS than viewing averted gaze (Pelphrey *et al.* 2004). Apart from the STS, the right MPFC has also been found to be activated when gaze is directed at, but not when gaze is averted away from, the self (Kampe *et al.* 2003; Schilbach *et al.* 2006). Interestingly, similar activation in the right MPFC has been reported when the person's name is called, indicating that communicative signals elicit common activations in this brain area, independent of their modality (Kampe *et al.* 2003).

**Figure 1:**
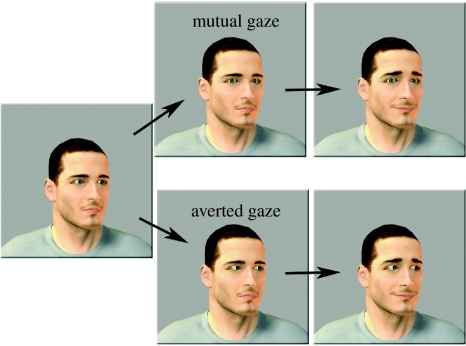
Still frames from the dynamic face stimuli used in both experiments. Note that gender, age and orientation of the face were randomly varied and counterbalanced. In the mutual gaze condition (upper half), the person's eyes moved towards the infant, and in the averted gaze condition (lower half), the person's eyes moved away from the infant. The eyebrow-raised and closed-mouth smiles were identical in the two conditions. Timing varied slightly between experiments 1 and 2 (see §2).

Our study investigated, using multiple neuroimaging techniques to allow good spatial and temporal resolution, which cortical regions are responsible for the perception of communicative cues in young infants. Four-month-old infants watched two kinds of dynamic communication scenarios in which a face either established mutual gaze or averted its gaze, both of which were followed by an eyebrow raise with accompanying smile (figure 1). Given the importance of face-to-face communication for human development and infants' early sensitivity to communication cues as evident in their behaviour (Farroni *et al.* 2002, 2003; Senju & Csibra 2008), we hypothesized that infants would show cortical activity differentiating between mutual and averted gaze during face-to-face communication in brain regions similar to those activated in adults.

So far, infant brain function has been predominantly investigated by using electroencephalography (EEG) and event-related potentials (ERPs), which offer good temporal but relatively poor spatial resolution. Thus, in experiment 1, we used near-infrared spectroscopy (NIRS), which permits more precise localization of brain activation by measuring haemodynamic responses. To date, this technique has been successfully used to study infant visual and language abilities (for a review, see Aslin & Mehler 2005). We used this technique to examine the cortical basis of face-to-face communication (with mutual versus averted gaze) in infants.

In experiment 2, we measured gamma-band oscillatory activity of the EEG in response to mutual and averted gaze cues in another group of four-month-old infants. Owing to the superior temporal resolution of the EEG, this allowed us to explore the exact timing of the cortical processes under investigation. (Note that although gamma-band EEG activity has a better temporal resolution than NIRS, ERPs offer an even better temporal measure. However, ERP components such as the face-sensitive infant N290 can only be easily measured in response to static stimuli, such as the initial presentation of a face, which did not differ across conditions in our study.) Moreover, gamma-band oscillations are of special interest because, consistent with a biophysical model (Kilner *et al.* 2005), they have been found to positively correlate with the haemodynamic response (Foucher *et al.* 2003; Niessing *et al.* 2005). We therefore predicted a good correspondence between haemodynamic responses as measured by NIRS (experiment 1) and gamma-band oscillations as measured by EEG (experiment 2).

## 2. Material and methods

### (a) Subjects

The final sample in experiment 1 consisted of 12 four-month-old infants (five girls) aged between 134 and 150 days (*M*=140 days, s.d.=5 days). An additional 10 four-month-olds were tested but not included in the final sample because they did not reach inclusion criteria (not enough artefact-free trials per condition (*n*=9) or too many missing channels (*n*=1)). The final sample in experiment 2 consisted of 12 four-month-old infants (six girls) aged between 136 and 148 days (*M*=142 days, s.d.=4 days). An additional 10 four-month-olds were tested but not included in the final sample because they did not reach inclusion criteria (not enough artefact-free trials per condition (*n*=10)). All infants were born full term (37–42 weeks gestation) and with normal birth weight (more than 2500 g).

### (b) Stimuli and procedure

Two experimental conditions were generated using the Poser v. 6.0 software (Curious Lab Inc., Santa Cruz, CA, USA). In each, infants viewed animated photorealistic faces with their heads oriented to the left or to the right (20°). In the mutual gaze condition (figure 1), a person's face appeared on the screen for 1000 ms, then the person moved her/his eyes towards the infant (100 ms gaze shift, without change in head orientation), where they remained for 900 ms, after which the person's expression changed from neutral into a closed-mouth smile plus eyebrow raise within 100 ms and then the person continued smiling for 900 ms. In experiment 1, the facial expression then changed back to neutral (duration: 1000 ms) and was followed by a second eyebrow raise and smile (duration: 1000 ms) while gaze direction remained constant. This additional eyebrow raise and smile was not presented in experiment 2 in order to reduce the stimulus presentation time and thus the number of eye movement artefacts that can affect the EEG signal more than the haemodynamic response measured in experiment 1. The averted gaze condition (figure 1) only differed from the mutual gaze condition in that the person on the screen moved her eyes away from the infant. We emphasize that, as in the adult work of Pelphrey *et al.* (2004), the size of the eye gaze shifts were identical across conditions, as were the timing of the dynamic events and the perceptual characteristics of the eyebrow raise and smile. Note also that prior to the gaze shift the two conditions were identical. Four different face identities (boy, girl, man and woman) were used. Infants sat on their parent's lap while watching the stimuli on a computer monitor within an acoustically shielded, dimly lit room. The visual angle was kept constant across experiments 1 and 2 although screens of different sizes were used. The faces presented subtended to 38×25°, and each eye subtended to 3×5°. A video camera centred on the infant's face allowed us to record infant gaze and behaviour. During baseline, infants' attention was drawn to the screen by a moving non-social stimulus (experiment 1: moving cars presented for 6 s; experiment 2: moving geometric shapes presented for randomly varying 500–700 ms). Face stimuli with mutual and averted gaze were presented in a pseudo-random order and with no more than two presentations of the same condition (mutual or averted) in a row, face identity changed from trial to trial, and face orientation (left or right) was counterbalanced. Infants who were included in the final sample completed an average of 21.3 trials (s.d.=4.5) in experiment 1 and 79.8 trials (s.d.=15.9) in experiment 2. This difference can be attributed to the difference in the length of trials including baseline (experiment 1, 15 s; experiment 2, 3.6 s).

### (c) Experiment 1: functional near-infrared spectroscopy

#### (i) Data acquisition and probe placement

To investigate cortical activation, NIRS measurements were made using the University College London topography system (Everdell *et al.* 2005). The multi-channel system uses two wavelengths at 770 and 850 nm in a frequency-multiplexed approach allowing rapid data acquisition. The arrays of channels are designed and adapted for each study protocol, allowing flexibility in the source detector geometry and locations of the arrays. In custom-built arrays and head gear, eight optodes in a 10-channel (source–detector pairs) arrangement with an inter-optode separation of 20 mm were placed over the temporal lobe on each hemisphere, and seven optodes in a six-channel (source–detector pairs) arrangement with the same separation were placed over the prefrontal cortex.

#### (ii) Data rejection

For each infant, the recorded near-infrared attenuation measurements were analysed and trials or channels were rejected from further analysis based on the quality of the signals. Criteria for channel rejection included the presence of large movement artefacts assessed by measuring the coefficient of variation (CV) of the signal. Channels were excluded if the CV of the attenuation measurement for each wavelength exceeded 10 per cent or if the difference in CV between the attenuation measurements for the two wavelengths (|CV770–CV850|) exceeded 5 per cent. These changes in CV could be due to the movement of the pad and hat, differential occlusion of the source fibres or a loose fibre in the pad. Channel rejection criteria also included high-frequency noise beyond the limits of physiological effects, where the normalized high frequency power is greater than 35 per cent of the total power of the signal (Blasi *et al.* 2007). Following conversion from attenuation to concentration data (see §2*c*(iii)), trials that contained changes of oxygenated haemoglobin (oxyHb) concentration that exceeded a predefined range (±3 μM during the prestimulus baseline (starting 4 s before face onset), and ±8 μM during the experimental trials (see also §2*c*(iii))) were removed from the dataset. In addition, infants' behaviour was coded from videotape using Supercoder software (Hollich 2005, Purdue University). Trials during which the infant did not fixate the screen for at least 90 per cent of the trial duration were excluded from further analysis. There was no difference in infants' behaviour (looking time, smiling or vocalization) between mutual and averted gaze conditions. The minimum number of valid trials per condition for each channel was seven; infants who were included in the final sample contributed on average 8.7 (s.d.=2.5) trials in the mutual and 8.3 (s.d.=2.1) trials in the averted condition. The minimum number of valid channels for each infant was seven for each of the temporal pads and four for the prefrontal pad; infants who were included in the final sample had an average of 17.1 (s.d.=2.3) valid channels in the two temporal pads and 5.2 (s.d.=1.9) valid channels in the prefrontal pads.

#### (iii) Data analysis

According to an established procedure (Blasi *et al.* 2007), for each infant, the signal was low-pass filtered, divided into 15 s blocks and detrended. Each block consisted of 4 s prior to the onset of the experimental trial non-social baseline (moving cars), the 5 s experimental face trial (mutual or averted gaze), followed by the 6 s non-social baseline (moving cars). The pre-processed attenuation data were then converted into changes in concentration of oxyHb and deoxygenated haemoglobin (deoxyHb) using the modified Beer–Lambert law and assuming a differential path-length factor for infants (Duncan *et al.* 1995). For each valid trial, the absolute peak of oxyHb or deoxyHb was determined within a time window from 2 to 10 s after face onset. This peak measure was then averaged across trials. Changes in oxyHb and deoxyHb were assessed statistically by comparing averaged peak amplitude measures between the two experimental conditions (mutual versus averted gaze) by using paired sample *t*-tests. The test values reported were corrected for multiple comparisons (Benjamini & Hochberg 1995; Benjamini & Yekutieli 2001). Please note that the degrees of freedom may differ slightly across channels because for individual infants no data were obtained from a particular channel. Effect size and power in experiments 1 and 2 were calculated with G^*^Power v. 3 (Faul *et al.* 2007).

### (d) Experiment 2: event-related gamma-band oscillations

#### (i) Measurement and data analysis

**Figure 2:**
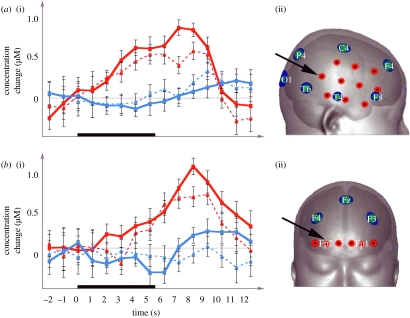
Haemodynamic responses (i) measured by NIRS in experiment 1; (*a*) right superior posterior temporal cortex and (*b*) right fronto-polar cortex. Red squares, oxyHb (mutual); red triangles, oxyHb (averted); blue squares, deoxyHb (mutual); blue triangles, deoxyHb (averted). The thick black line on the time axis represents the duration during which the face stimuli were presented. (ii) NIRS channel layout (red circles) is shown on scalp surface with reference to a 10–20 system of EEG electrode placement (blue circles represent electrode positions) and approximate underlying cortical structures (Okamoto *et al.* 2004). Channels for which the time course is presented are marked by an arrow.

The brain electrical activity was recorded using a Geodesic Sensor Net consisting of 128 electrodes evenly distributed across the scalp and the vertex lead serving as a reference. The electrical potential was amplified with 0.1–200 Hz bandpass, digitized at 500 Hz sampling rate. Artefacts caused by eye and body movements were eliminated by manual rejection. In addition, the infants' visual behaviour was coded from videotape, and trials during which the infant did not fixate the screen during stimulation were excluded from further analysis. Participants who were included in the final sample contributed at least 20 trials per condition (mean number of trials: 28.3 (s.d.=7.6) for mutual gaze and 29.4 (s.d.=8.2) for averted gaze). Induced gamma oscillations were analysed using an established procedure (Grossmann *et al.* 2007) in which we applied a continuous wavelet transformation to single trials of EEG in each channel, using Morlet wavelets at 1 Hz intervals (20–90 Hz). The EEG data were re-referenced to average reference before the wavelet transformation. The wavelet transformation was performed on 3700 ms long EEG segments (500 ms prestimulus onset and 1000 ms post-stimulus onset). EEG data for 200 ms at the beginning and at the end of each segment had to be removed due to the distortion in the time–frequency decomposition commonly caused by wavelets. The average amplitude during a 200 ms prestimulus interval 100 ms prior to the stimulus onset (−300 to −100) was considered as the baseline and was subtracted from the whole time-varying signal. In order to rule out that the effects observed were due to differences before stimulus onset, we also tested whether there were any statistical differences between conditions during the baseline period (−300 to −100 ms), which was tested by comparing the baseline activity between conditions before we applied the baseline correction. This comparison revealed no difference between conditions during baseline. We examined the mean amplitude in 200 ms blocks to assess statistically the amplitude of the gamma oscillation between 30 and 50 Hz. This frequency range of approximately 40 Hz was chosen on the basis of previous adult and infant work (Tallon-Baudry & Bertrand 1999; Grossmann *et al.* 2007), and visual inspection of the data (see figure 1 in the electronic supplementary material) indicated that the effects were restricted to this range. We constrained our analysis of the gamma band spatially by defining regions of interest according to the brain locations identified in experiment 1 (NIRS; these regions of interest were consistent with the topography of the gamma-band effects observed; see figure 2 in the electronic supplementary material). Thus, paired sample *t*-tests were conducted for four different scalp locations (left and right fronto-polar regions and left and right posterior superior temporal regions).

## 3. Results

### (a) Experiment 1: functional near-infrared spectroscopy

**Figure 3:**
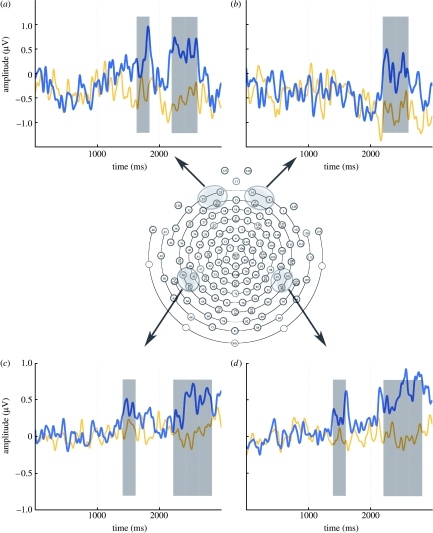
Amplitude of gamma-band (30–50 Hz) activity (in μV) plotted over time (in ms) in the mutual (blue) and averted gaze (orange) condition. Time windows during which the two conditions differed significantly from each other are marked in grey. The eye gaze shift occurred at 1000 ms and the eyebrow raise with smile at 2000 ms. (*a*) Left fronto-polar, (*b*) right fronto-polar, (*c*) left posterior temporal, and (*d*) right posterior temporal.

Our analysis (see §2) of infants' haemodynamic brain responses revealed two brain regions that were sensitive to dynamic gaze direction: the right superior posterior temporal cortex (figure 2*a*) and the right fronto-polar cortex (figure 2*b*). These two brain regions showed significant increases in oxyHb concentration both when the mutual gaze condition was contrasted to the baseline (moving car), where no social stimuli were present (right superior posterior temporal cortex: *t*(11)=3.37, *p*=0.006; right fronto-polar cortex: *t*(10)=3.02, *p*=0.013), and when the mutual gaze condition was compared with the averted gaze condition (right superior posterior temporal cortex: *t*(11)=2.29, *p*=0.043, sign test, *p*=0.039; right fronto-polar cortex: *t*(10)=2.83, *p*=0.018, sign test, *p*=0.012; see figure 3 in the electronic supplementary material for comparisons between mutual and averted gaze at all channels). Effect sizes (Cohen's *d*) for all findings reported above were greater than 0.84 and calculated power values were greater than 0.91. The corresponding brain regions in the left hemisphere also showed an increase in oxyHb concentration that was greater in the mutual gaze condition than in averted gaze condition and during the baseline, but these differences failed to survive the correction for multiple comparisons. In addition, we assessed the mean latency (in seconds) of the maximum peak of oxyHb concentration with respect to the following face onset: (i) right superior posterior temporal cortex (mutual: *M*=6.73 s (s.e.=0.85 s); averted: *M*=6.44 s (s.e.=1.62 s)) and (ii) right fronto-polar cortex (mutual: *M*=7.81 s (s.e.=0.93 s); averted: *M*=7.98 s (s.e.=1.31 s)). This analysis revealed no statistical differences in latency between the conditions and brain regions. The peak latencies observed in the current infant study are consistent with those generally reported in the literature for adults (Buckner 2002). No brain regions were found in which the oxyHb concentration changes were higher in the averted than the mutual gaze condition, and the analysis of deoxyHb concentration changes revealed no significant differences between conditions. The fact that we did not find any significant decreases in deoxyHb that accompanied the increase in oxyHb, as one would expect on the basis of adult work (Obrig & Villringer 2003), is in line with previous infant NIRS work (Lloyd-Fox *et al.* in press; Nakato *et al.* in press). These infant NIRS studies either failed to find a significant decrease or even observed an increase in deoxyHb concentration. Although a number of factors such as immaturity of the infant brain have been suggested to explain this difference between infants and adults, the exact nature of this difference remains an open question (for a discussion, see Karen *et al.* (2008) and Nakato *et al.* (in press)).

### (b) Experiment 2: event-related gamma-band oscillations

Our analysis of infants' gamma-band oscillatory brain activity (30–50 Hz), which was topographically constrained based on the NIRS findings in experiment 1 (see §2), revealed that gamma activity increased in the context of mutual gaze. As shown in figure 3, we assessed the time course of the neural activity changes and were able to identify which dynamic change in the face (gaze shift at 1000 ms or eyebrow raise and smile at 2000 ms) induced changes in the gamma activity. This analysis revealed that, whereas at bilateral posterior temporal and left fronto-polar regions two significant bursts of gamma-band activity were observed, one in response to mutual gaze shift (right posterior temporal region (1400–1600 ms): *t*(11)=2.77, *p*=0.018; left posterior temporal region (1400–1600 ms): *t*(11)=2.64, *p*=0.023, *p*=0.006; left fronto-polar region (1600–1800 ms): *t*(11)=2.59, *p*=0.015), and one to the eyebrow raise and smile when it followed mutual gaze (right posterior temporal region (2200–2800 ms): *t*(11)=3.16, *p*=0.009; left posterior temporal region (2200–2800 ms): *t*(11)=2.84, *p*=0.016; left fronto-polar region (2200–2600 ms): *t*(11)=2.96, *p*=0.013), the right fronto-polar region showed an increase of activity only in response to the eyebrow raise and smile in the context of the mutual gaze (right fronto-polar region (2200–2600 ms): *t*(11)=2.88, *p*=0.015). Effect sizes (Cohen's *d*) for all findings reported above were greater than 0.87 and calculated power values were greater than 0.79.

## 4. Discussion

We adopted a novel approach by measuring haemodynamic responses with NIRS (experiment 1) and gamma oscillatory activity with EEG (experiment 2) to examine the neural bases of non-verbal social communication in four-month-old infants. The results revealed that mutual gaze activates cortical areas in the infant brain that correspond to the brain regions implicated in these processes in adults (Kampe *et al.* 2003; Pelphrey *et al.* 2004). In addition, EEG analysis (experiment 2) revealed that mutual gaze itself, and the eyebrow raise with an accompanying smile in the context of mutual gaze, produce similar cortical activations. The fact that the eyebrow raise with an accompanying smile generates this activation only if mutual gaze has already been established demonstrates that these brain responses reflect the interpretation of the eyebrow raise and smile as a communicative signal rather than merely being elicited by the physical change of the stimulus.

Our analysis of infants' haemodynamic brain responses (experiment 1) revealed two brain regions that are sensitive to dynamic gaze direction, as reflected in increased oxyHb concentration when the mutual gaze condition was compared with the averted gaze condition. These regions were (i) right superior posterior temporal cortex and (ii) right fronto-polar cortex. We now discuss these in turn.

Functionally, the superior posterior temporal cortex is thought to play an important role in biological motion processing and has been consistently activated in response to eye gaze and facial expressions in adults (Allison *et al.* 2000). Our results with infants are consistent with findings of a similar fMRI experiment with adults, which indicated that mutual gaze, when compared with averted gaze, results in increased activity in this brain region (Pelphrey *et al.* 2004). It is important to note that in adults this area is not specifically tuned to one particular gaze direction but its activity is modulated by the social context in which a gaze shift occurs (for a discussion, see Pelphrey & Morris 2006). As in adults, this effect was lateralized to the right hemisphere in infants, suggesting that right hemisphere dominance in these kinds of processes develops early in ontogeny. However, note that neither in the prior adult fMRI work (Pelphrey *et al.* 2004) nor in the current infant study was this effect absent in the left hemisphere.

The MPFC, apart from having various other functional roles in ‘mentalizing’ (Amodio & Frith 2006), is also concerned with reading communicative intentions, and mutual gaze and calling the person's name have been found to consistently activate this region in adults (Frith 2007). Human communication conveys not only the message to be delivered between sender and receiver but also depends critically upon the initial detection of the intention to communicate (Grice 1969; Searle 1969). Establishing eye contact with the intended recipient is often used to fulfil this function. In experiment 1, we found that mutual gaze resulted in increased activity within a similar prefrontal region in infants as that which is activated by communication cues in adults. Our current measurement technique does not allow us to assess at which depth the source of this activation is located (more superficial or deeper into the medial aspects of the prefrontal cortex). Nevertheless, the functionally similar brain responses make it likely that the adult and infant neuroimaging results represent homologous brain processes.

The finding that across experiments, and even within subjects, neural activity and haemodynamic responses show corresponding functional effects (see figure 4 in the electronic supplementary material for additional results) can be seen as a cross-validation of methods used. Comparing the findings from experiments 1 and 2 seems to suggest different lateralization patterns in brain activity, with the gamma activity indicating a broader distribution across both hemispheres. However, as noted above, the haemodynamic differences measured by NIRS, although lateralized, were not restricted to the right hemisphere. Clearly, future work will be needed to more directly explore neurovascular coupling (Obrig & Villringer 2003) by combining NIRS and EEG measurements in the same infant co-registered against structural MRI data.

The pattern of results that we obtained in this study suggests an early specialization of the cortical regions involved in the perception of facial communication cues, and contrasts with findings indicating a much more gradual specialization of the recognition system that deals with invariant aspects of faces (de Haan *et al.* 2002; Cohen-Kadosh & Johnson 2007; but see Tzourio-Mazoyer *et al.* 2002). With regard to the functional organization of the face-processing system in humans, these differential developmental trajectories of cortical specialization provide further indirect evidence for a distinction between these two aspects of face processing, and thus support existing models of face perception (Haxby *et al.* 2000, 2002). The earlier cortical specialization for facial communication may be explained by the pivotal adaptive role that social interaction plays very early in human development and learning (Csibra & Gergely 2006; Kuhl 2007). Correspondingly, the more protracted specialization of the face recognition system might point to a greater dependence on experience and perceptual learning—a process that may even be influenced by the biases introduced by the more precocious system for facial communication (Gliga & Csibra 2007). However, since our study did not compare these two aspects of face perception directly, our conclusion about the décalage of these neural systems remains tentative.

Our results also demonstrate for the first time that four-month-old infants can detect mutual gaze even when a person's head is oriented to the side. This is a difficult task even for adults because it requires the integration of eye and head orientation (Pageler *et al.* 2003). In previous work using static face stimuli, we found that four-month-olds produced prefrontal gamma activity in response to mutual gaze only in the context of a canonical frontal face but not when the head was oriented to the side (Grossmann *et al.* 2007; Grossmann & Farroni in press). This suggests that the dynamic cues provided in the current study might have helped the infants to extract the communicative information conveyed by mutual gaze.

The nature and development of human social cognitive abilities is currently hotly debated (Frith & Frith 2007; de Waal *et al.* 2008). For example, a recent study reported that apes and 2.5-year-old children have very similar cognitive skills when it comes to dealing with the physical world, but that children have more sophisticated skills than apes for dealing with the social world (Herrmann *et al.* 2007). A fundamental issue concerns whether these human-specific abilities of engaging with others are the product of our intensely social early environment or, as some have hypothesized, that humans are uniquely adapted for collaborating with and learning from others from early in life (Tomasello *et al.* 2005; Csibra & Gergely 2006). In support of the latter view, our study demonstrates very early specialization of the brain regions that process face-to-face social interaction in young human infants, including a region in prefrontal cortex with human-specific anatomical and functional properties (Semendeferi *et al.* 2001; Allman *et al.* 2002; Saxe 2006).
