# Supplementary material for: Early cortical specialization for face-to-face communication in human infants
Source: Proc Biol Sci. 2008 Aug 28;275(1653):2803–11. doi: 10.1098/rspb.2008.0986 (PMC2572680; doi:10.1098/rspb.2008.0986)
Supplement: Supplementary file 1 [file rspb20080986s02.doc]

**ELECTRONIC SUPPLEMENTARY MATERIAL**

**Figure 1:** Example of time-frequency plot of the gamma-band EEG responses to averted and mutual gaze at left fronto-polar region (see Methods for channel selection). As shown here, the gamma-band response was confined to frequencies from 30-50 Hz and two bursts of gamma activity were observed: one in response to mutual gaze shift and another in response to the smile accompanied by the eyebrow raise.
