# Supplementary material for: Early cortical specialization for face-to-face communication in human infants
Source: Proc Biol Sci. 2008 Aug 28;275(1653):2803–11. doi: 10.1098/rspb.2008.0986 (PMC2572680; doi:10.1098/rspb.2008.0986)
Supplement: Supplementary file 2 [file rspb20080986s03.doc]

**ELECTRONIC SUPPLEMENTARY MATERIAL**

**Figure 2:** Illustration of the scalp topography of the EEG gamma-band effects (30-50 Hz; 2300 to 2360 ms) for both experimental conditions in Experiment 2 (top: front of the head; bottom: back of the head). Marked in black are the channel positions at the four regions of interest (left and right fronto-polar, left and right posterior temporal) that were used for analysis (see Methods).
