# Supplementary material for: Early cortical specialization for face-to-face communication in human infants
Source: Proc Biol Sci. 2008 Aug 28;275(1653):2803–11. doi: 10.1098/rspb.2008.0986 (PMC2572680; doi:10.1098/rspb.2008.0986)
Supplement: Supplementary file 3 [file rspb20080986s04.doc]

**ELECTRONIC SUPPLEMENTARY MATERIAL**

**Figure 3:** The top panel illustrates the NIRS channel layout used in Experiment 1 shown on scalp surface. Each channel is marked in red and has a number. To provide an illustration of the spatial distribution of the effects, for each of the channels, results from a paired t-test (mutual minus averted gaze) are presented in the tables below (* indicates significance level of < 0.05). Please note that for the lateral pads the channel numbers presented here only for the right hemisphere refer to anatomically corresponding locations in the left hemisphere.
