# Supplementary material for: Early cortical specialization for face-to-face communication in human infants
Source: Proc Biol Sci. 2008 Aug 28;275(1653):2803–11. doi: 10.1098/rspb.2008.0986 (PMC2572680; doi:10.1098/rspb.2008.0986)
Supplement: Supplementary file 4 [file rspb20080986s05.doc]

**ELECTRONIC SUPPLEMENTARY MATERIAL**

**Figure 4:** To assess the relationship between hemodynamic measures (NIRS) and gamma band responses (EEG) within subjects, four infants who participated in Experiment 1 were also tested 2-3 weeks later using the EEG paradigm (Experiment 2). In order to examine this relationship statistically we calculated a difference measure between the peak responses in oxyHb and the gamma band in the two experimental conditions (mutual minus averted) for the brain regions identified in Experiment 1. For these infants we found a positive correlation between the hemodynamic signal change and the gamma band difference (*r* = 0.61; *p* = 0.012). In order to illustrate the positive correlation between the two measures, the differences of the peak responses in oxyHb in μM (x- axis) and the gamma band in μV (y-axis) between the two experimental conditions (mutual minus averted) were calculated and are plotted here for four brain regions (left and right posterior temporal regions, left and right prefrontal regions).
